# Meeting Abstracts from the 70^th^ Annual British Thyroid Association Meeting: London, UK. 20^th^ May 2022

**DOI:** 10.1186/s13044-022-00129-1

**Published:** 2022-07-22

## L1 George Murray Lecture: Occam’s razor, Bayes’ theorem and the practice of thyroidology in a cold climate

### Petros Perros (petros.perros@newcastle.ac.uk)

#### Royal Victoria Infirmary, Newcastle upon Tyne, UK

Following George Murray’s discovery of the cure for myxoedema, levothyroxine (LT4) substituted thyroid extract and until the late 20th century, the treatment of hypothyroidism with LT4 was generally regarded as simple, effective and easy. Patient dissatisfaction with LT4 emerged in the 21st century. The academic community responded with an unprecedented number of randomised controlled trials comparing LT4 with liothyronine (LT3)-containing treatments, which failed to confirm superiority. Despite the lack of evidence clinicians have increasingly prescribed LT3, usually responding to patient demands. In 2022 we are faced with a worrying rising trend for overdiagnosis of hypothyroidism, use of thyroid hormones in euthyroid patients and overtreatment with thyroid hormones. Evidence from recent surveys reveals widespread dissatisfaction among hypothyroid patients with their treatment and care; the majority of endocrinologists are unconvinced about the superiority of LT3-containing treatments, yet they seem to prescribe it to patients who request it as a remedy for persistent symptoms. The survey data also revel a remarkable geographic variation in the self-reported prevalence of persistent symptoms among hypothyroid patients with northwest Europe and north America having the highest prevalence. The extraordinary academic endeavour and focus on LT3-containing treatments as a means for addressing persistent symptoms, is contrasted by the almost total neglect of other likely contributors, most notably medically unexplained symptoms. Evidence is now beginning to emerge that non-pharmacological approaches to the conundrum of persistent symptoms among treated hypothyroid patients, may be effective.

## S1 Hyperthyroidism before and during pregnancy

### Stine Linding Andersen^1,2^ (stine.a@rn.dk)

#### ^1^Department of Clinical Biochemistry, Aalborg University Hospital, Aalborg, Denmark; ^2^Department of Clinical Medicine, Aalborg University, Aalborg, Denmark

Hyperthyroidism in women of fertile age is a special clinical case. The management of these patients should consider the possibility of a current or future pregnancy, and in pregnancy the management should ensure the health of the pregnant woman, but also the development of the fetus. Fetal development is particularly prone to any adverse exposures in the early pregnancy when the organs are formed, and management of maternal hyperthyroidism in this critical time window is particularly important. At the same time, maternal physiology is markedly affected by the pregnant state from the early pregnancy weeks which challenges the biochemical assessment of maternal thyroid function and the differential diagnosis of Graves’ hyperthyroidism as opposed to gestational hyperthyroidism. Furthermore, physiological alterations in the immune system in and after a pregnancy may alter disease activity and the need for treatment. On the tip of the iceberg, the clinical management is challenged by the risk of teratogenic side effects associated with the current available antithyroid drugs. Hyperthyroidism in young female patients has been a matter of raised clinical awareness for decades, but uncertainty prevails on the underlying mechanisms and the role of the different exposures associated with the disease (hyperthyroidism *per se*, autoimmunity, and antithyroid drug treatment). Evidence to support outcomes of the different exposures associated with maternal hyperthyroidism include experimental findings, human case reports and case series, as well as large observational cohort studies. Methodological aspects and study differences challenge the current state of the art and hold important implications to consider as part of future scientific work. More evidence is needed to inform the clinical practice guidance on the management of the disease before and during pregnancy.

## S2 Thyroid management during assisted reproduction

### Kris G. Poppe (kpoppe@ulb.ac.be)

#### Department of Internal Medicine – Endocrine Unit, University Hospital St-Pierre Brussels, Brussels, Belgium

Severe thyroid dysfunction may lead to menstrual disorders and infertility via direct and indirect interactions with the hypothalamo-pituitary-ovarian axis and the reproductive organs. However, the exact prevalence of infertility in women with thyroid disorders remains unknown. Fertility problems may persist even after restoring normal thyroid function, and then surgery and/or an assisted reproductive technology (ART) may be necessary to obtain a pregnancy. The initial step in an ART treatment is the ovarian stimulation, putting strain on the thyroid gland, potentially leading to (permanent) hypothyroidism in women with thyroid autoimmunity (TAI) or when already treated with thyroid hormones (LT4). Moreover, women with ovarian and unexplained causes of infertility have a higher prevalence of TAI. In women treated with LT4, a serum TSH level <2.5 mIU/L should be targeted before ART. In women with TSH levels >4.0 mIU/L, fertilisation rates, embryo quality and live birth rates may be impaired, but also improved with LT4 therapy. In euthyroid women with serum TSH >2.5 mIU/L and TAI, LT4 should not be given systematically, but on a case-by-case basis. For all of the above reasons, women of infertile couples should be screened routinely for the presence of thyroid disorders. The importance of the increased use of intracytoplasmic sperm injection as a type of ART on pregnancy outcomes in women with TAI deserves more investigation. In this presentation, we will focus on recent ETA-guidelines, but also on the gaps in the current knowledge, the remaining questions on the associations between thyroid (disorders) and (assisted) reproduction.

## S3 Hypothyroidism and thyroid autoimmunity in pregnancy

### Rima Smith-Dhillon (r.k.smith@bham.ac.uk)

#### University of Birmingham, Birmingham, UK

Pregnancy loss is a devastating event for couples. Miscarriage is defined as the loss of a pregnancy before viability and the average risk of miscarriage is 15%. Hypothyroidism (overt and subclinical) is a common condition associated with adverse pregnancy outcomes, such as miscarriage, if untreated. In addition thyroid autoimmunity, even in euthyroid women, has repeatedly been associated with negative pregnancy outcomes, such as miscarriage and preterm birth. There is international debate in medicine about whether there should be routine testing of thyroid function in individuals who are trying for a baby, in view of the effect of thyroid dysfunction on pregnancy. In addition, the strategies used to manage certain thyroid problems are questioned. Discussions around testing and subsequent management particularly relate to women with a history of repeated miscarriages.

Overt hypothyroidism is treated with levothyroxine therapy and evidence has demonstrated benefit with this treatment when initiated preconception or in pregnancy. However, while it is almost universally agreed that subclinical hypothyroidism (SCH) should also be treated with levothyroxine, the evidence of its benefit is less clear. The two largest randomised controlled trials (RCT’s) on the subject found no improvement in obstetric or neonatal outcomes with levothyroxine treatment in pregnancy; however the treatment was not initiated until early 2^nd^ trimester which is thought to be too late. In addition, there is debate over what cut-off of thyroid stimulating hormone (TSH) should be applied when making the diagnosis of SCH. Thyroid autoimmunity is prevalent in around 9% of women of reproductive age. Evidence from high quality RCT’s has shown that empirical treatment of euthyroid TPOAb positive women does not improve pregnancy outcomes; this includes when specifically looking at women with recurrent pregnancy loss. Therefore, routine treatment of levothyroxine in euthyroid TPOAb positive women cannot be recommended.

## S4 The year in benign thyroid disease research

### Fraser Gibb (fraser.gibb@ed.ac.uk)

#### Edinburgh Centre for Endocrinology & Diabetes, University of Edinburgh*,* Edinburgh, UK

Hypothyroidism:

A small RCT comparing levothyroxine and liothyronine (LT4+LT3) vs. desiccated thyroid extract (DTE) vs. LT4 did not find any difference in quality of life, thyroid symptoms or patient preference. Although, subgroup analysis hinted that those with greater symptoms at baseline may favour LT3 containing therapies (10.1210/clinem/dgab478).

A US study did not detect any major effects upon thyroid function tests (TFTs) in people switching between generic LT4 preparations (10.1001/jamainternmed.2022.0045).

Differences in two of the most commonly used TFT assays in the UK are likely to result in clinically important differences in the diagnosis of subclinical hypothyroidism (10.1111/cen.14423).

Graves’ disease:

The PRAGMA study has provided important data on outcomes following radioiodine therapy for Graves’ disease.

Statins and thyroid eye disease:

Observational data (10.1210/clinem/dgab070) and RCT evidence (10.1016/S2213-8587(21)00238-2) suggest that statins can be repurposed to improve outcomes in Graves’ orbitopathy.

Diabetes risk in LT4 replaced individuals:

People treated with thyroidectomy for thyroid cancer in South Korea had significantly increased rates of incident type 2 diabetes (10.1210/clinem/dgab776). Similar findings were reported in a meta-analysis (10.1210/clinem/dgac006) and observational study of thyroid disease in adolescents (10.1210/clinem/dgab382).

Covid and thyroid:

Thr92Ala-DIO2 polymorphism was associated with differences in mortality in Covid-19 patients (10.1210/clinem/dgac075). Covid-19 and Covid vaccines have also been associated with incident thyrotoxicosis (10.1210/clinem/dgac119 & 10.1210/clinem/dgac049).

## S5 The year in basic thyroid research

### Krishna Chatterjee (kkc1@medschl.cam.ac.uk)

#### Wellcome-MRC Institute of Metabolic Science, Box 289, Addenbrooke’s Hospital, Cambridge, UK

**Background**: Basic thyroid research can advance understanding of normal thyroid physiology and inform the basis or management of thyroid disease.

**Methods**: A review of published literature in 2021-22, focussed on reports fulfilling the above criteria.

**Results**: The *SLC17A4* genomic locus is associated with circulating FT4 concentrations (Teumer et al, Nature Comms 2018). Now, studies indicate that SLC17A4, expressed in non-neuronal tissues, is a T3/T4 transporter (Groeneweg et al Thyroid 2022).

Deficiency of the MCT8 thyroid hormone transporter causes severe intellectual and motor disability (Groeneweg et al Lancet Diabetes & Endo 2020). Administration of adenovirally-expressed MCT8 to Mct8/Oatp1c1 double knockout mice, nearly normalises brain T3 content and increases thyroid hormone (TH)-dependent gene expression (Liao et al Thyroid 2022).

Deiodinase enzymes regulate the availability of T3 - the active TH. In the first report of inherited type 1 deiodinase (*DIO1)* deficiency in humans, two, missense *DIO1* mutations in unrelated families are associated with abnormal TH metabolism (raised rT3 and rT3/T3 ratios) (Franca et al Thyroid 2022).

Genes involved in cell signalling (e.g. *PTEN*, *APC*) or RNA processing (*DICER*) can cause thyroid cancer. Mutations in *WDR77*, a new cause of familial papillary thyroid cancer, cause altered histone methylation and cell proliferation (Zhao et al PNAS 2021), possibly representing a new oncogenic pathway in thyroid cancer.

Teprotumumab, an insulin-like growth factor 1 receptor (IGF-1R) blocking antibody, is approved for the treatment of thyroid eye disease (Douglas et al, NEJM 2020) and effective in thyroid dermopathy (Crespo-Trevino et al, EDMCR 2022). An anti-TSHR antibody activates IGF-1R signalling in human fibroblasts; an anti-IGF-1R antibody suppresses this, causing apoptotic cell death. Such IGF-1R/TSHR crosstalk, provides a mechanistic basis for the efficacy of teprotumumab (Morshed et al Thyroid 2022).

**Conclusions**: Recent scientific advances in thyroid signalling, cancer and autoimmunity, of potential clinical significance, have been highlighted.

## S6 The year in thyroid cancer research

### Kate Newbold (knewbold@nhs.net)

#### The Royal Marsden NHS Foundation Trust, London, UK

This talk will review emerging data over the last few years which have led to changes in the management of thyroid cancer. It will cover early stage differentiated cancer and discuss evidence determining the benefit of radioiodine; update on the role of re-differentiation for continued therapy with radioiodine; review systemic therapies for iodine refractory thyroid cancer; discuss exciting advances in the management of anaplastic thyroid cancer with targeted and immunotherapies and the evidence for and when specific RET inhibitors should be considered for medullary thyroid cancer. It will highlight and signpost recent guideline updates and innovative practice aids such as TIRO (Thyroid International Recommendations Online).

## OR1 Targeting the TSH receptor with human monoclonal autoantibody K1-70TM – outcomes of a phase 1 clinical trial

### J Furmaniak^1,2^, J Sanders^1,2^, P Sanders^2^, Y Li^2^, B Rees Smith^1,2^

#### ^1^AV7 Limited, FIRS Laboratories, Parc Ty Glas, Llanishen, Cardiff, CF14 5DU, UK; ^2^FIRS Laboratories, RSR Limited, Parc Ty Glas, Llanishen, Cardiff, CF14 5DU, UK

##### **Correspondence:** J Furmaniak (firs@rsrltd.eclipse.co.uk)

**Background and Aims:** Stimulation of the TSH receptor (TSHR) by TSHR autoantibodies (TRAb) has a key role in the pathogenesis of Graves’ disease (GD) and Graves’ orbitopathy (GO). K1-70TM is a human monoclonal autoantibody which binds to the TSHR and prevents TSHR stimulation by TSH and TRAb and we now describe results of a K1-70 TM phase 1 clinical trial.

**Methods:** Safety, tolerability, pharmacokinetic, pharmacodynamics and immunogenic effects of K1-70TM in patients with GD were assessed. Patients received ascending doses of K1-70TM in 6 cohorts of 3 subjects each. K1-70TM doses from 0.2 mg to 25 mg were administered intramuscularly (im) and 50mg or 150 mg intravenously (iv). All subjects completed a 100 day follow up.

**Results:** K1-70TM was well tolerated in all subjects at all doses. There were no deaths or Serious Adverse Events. Adverse Events were mild or moderate and not directly related to K1-70TM. There were no immunogenic responses. Administration iv resulted in improved systemic exposure compared to im indicating iv was the correct dosage route. Following higher doses (25 mg and above) of K1-70TM serum fT3, fT4 and TSH progressed into hypothyroid ranges. On day 28 post dose 11/18 (61%) of patients were hypothyroid with all 9 receiving 25mg or more becoming hypothyroid on or before day 28. This corresponded with improvements in the signs and symptoms of GD and GO. Significant reductions in exophthalmos (> 2 mm) were recorded in subjects receiving higher doses of K1-70TM.

**Conclusions:** K1-70TM was safe and well tolerated in all subjects. The pharmacokinetic/pharmacodynamics relationship exceeded expectations of the phase 1 trial and K1-70TM shows considerable promise as a new drug to control TSHR activity in patients with GD and GO.

## OR2 Repurposing of disulfiram and diethyldithiocarbamate (DDC)-metal complexes to enhance NIS function in radioiodide therapy

### Kate Brookes^1^, Ling Zha^1^, Mehjabi Moolla^1^, Jana Kim^2^, Merve Kocbiyik^1^, Selvambigai Manivannan^1^, Hannah R Nieto^1^, Vinodh Kannappan^3,4^, Weiguang Wang^3,4^, Kavitha Sunassee^2^, Philip J Blower^2^, Vicki E Smith^1^, Martin L Read^1^, Christopher J McCabe^1^

#### ^1^Institute of Metabolism and Systems Research, University of Birmingham, Birmingham, UK; ^2^School of Biomedical Engineering & Imaging Sciences, King’s College London, London, UK; ^3^Research Institute in Healthcare Science, Faculty of Science and Engineering, University of Wolverhampton, Wolverhampton, UK; ^4^Disulfican Ltd, University of Wolverhampton Science Park, Wolverhampton, UK

##### **Correspondence:** Kate Brookes (K.Baker.2@bham.ac.uk)

**Background:** New drug approaches are urgently needed that enhance radioiodide (RAI) uptake leading to efficient ablation of thyroid cancer cells, especially in RAI-refractory disease. We recently utilised high throughput screening and identified FDA-approved compounds capable of inducing sodium iodide symporter (NIS) function to enhance iodide uptake, including the proteasomal/VCP inhibitor disulfiram. In vivo, disulfiram is rapidly metabolized to diethyldithiocarbamate (DDC), which binds metal ions (e.g. copper or zinc), and is currently being investigated for use in wide-ranging therapeutic applications including cancer and parasitic infection.

**Aims:** To gain a mechanistic understanding of how disulfiram and its related DDC metal complexes (e.g. Cu(DDC)2) impact NIS function in thyroid cells in vitro and in vivo.

**Methods:** NIS function was monitored in cultured cells by RAI (125I) uptake assays. Technetium-99m pertechnetate (99mTc) uptake after intraperitoneal administration was used to evaluate NIS function in Cu(DDC)2-treated Balb/c mice.

**Results:** Disulfiram, as well as DDC-metal complexes such as Cu(DDC)2, induced significant NIS protein expression and 125I uptake (up to 8-fold; 100-500 nM; P<0.001) in multiple thyroid cell types, including human primary thyrocytes. Importantly, disulfiram and Cu(DDC)2 retained the ability to enhance NIS function in thyroid cells ablated for expression of either VCP or its co-factor NPL4, indicating their effect on NIS was via VCP-independent pathways. Instead, a transcriptional effect of Cu(DDC)2 was revealed by significant induction in NIS mRNA levels in thyroid TPC-1 (8.5-fold) and 8505C (104.8-fold) cells. Similarly, Cu(DDC)2 induced the mRNA expression of other thyroid-specific genes such as thyroglobulin (6.1-fold). In vivo, Cu(DDC)2 enhanced thyroidal uptake of 99mTc at 30 min post-administration (~46.6%; n=5 per group; 3 mg/kg dose; P=0.0095), confirming significant induction of NIS function.

**Conclusions:** These results demonstrate that disulfiram and its related DDC-metal complexes represent a promising drug strategy to modulate NIS function with real clinical potential to enhance radioiodide therapy.

## OR3 Structures of Human Thyroid Peroxidase (TPO) in Complex with TPO Antibodies determined by Cryo-electron Microscopy

### S Baker, R Núñez Miguel, D Thomas, M Powell, J Furmaniak, B Rees Smith

#### FIRS Laboratories, RSR Ltd, Parc Ty Glas, Llanishen, Cardiff, CF14 5DU, UK

##### **Correspondence:** J Furmaniak (firs@rsrltd.eclipse.co.uk)

**Background and Aims:** Thyroid peroxidase (TPO) is a key enzyme in the synthesis of thyroid hormones and a major autoantigen in autoimmune thyroid disease. TPO autoantibodies (TPOAb) have been reported to bind to the epitopes on the peroxidase domain (POD) and a complement control protein like domain (CCP). The structure of TPO bound to antibodies has now been determined using cryo-electron microscopy (cryo-EM).

**Methods:** The extracellular domain (ECD) of human TPO (amino acids; aa 1-839) was expressed in insect cells and complexed with a TPO human monoclonal autoantibody 2G4 (Fab) or a TPO mouse monoclonal antibody 4F5(Fab). Cryo-EM was performed on a Titan Krios 300kV with a Falcon 3 Direct Detector.

**Results:** The structures of TPO-2G4 and TPO-4F5 complexes were solved at 3.92Å and 3.4Å resolution respectively. The structure shows the TPO ECD comprising the POD, the CCP and an incomplete epidermal growth factor like domain (EGF). The haem group of POD is held by Arg396 and Arg491 which form salt bridges with two carboxylate groups of the haem. His494 interacts with the iron ion of the haem. A calcium ion is coordinated by Asp240, Thr321, Phe323, Asp325, and Ser327. The enzyme active site is lined by Gln235, Asp238, His239 and Glu399.

Both antibody epitopes are located exclusively on the POD. 2G4 epitope comprises aa 194-277 and 604-628 whereas 4F5 aa 461-659, with three common residues Glu604, Ala607 and Asp608 for both epitopes. The CCP and EGF do not contribute to the antibody binding epitopes. A disulphide bond between POD Cys768 and CCP Cys794 would prevent any conformational movement of the CCP or EGF towards the antibody epitopes on the POD.

**Conclusions:** The molecular structure of TPO has been solved. This should be helpful in improving our understanding of thyroid autoimmunity and developing effective inhibitors of TPO enzyme activity.

## OR4 Using genetics to test whether umbilical cord FT4 or TSH levels are causally related to birthweight

### Brandon Lim^1^, Jessica Tyrrell^1^, Robin N Beaumont^1^, Beverley Shields^1^, M. Carolina Borges^2^, Andrew Hattersley^1^, Nicole Warrington^3^, David Evans^3^, Bijay Vaidya^1^, Deborah A. Lawlor^2^, Rachel M. Freathy^1^

#### ^1^University of Exeter, Exeter, UK; ^2^University of Bristol, Bristol, UK; ^3^University of Queensland, Australia

##### **Correspondence:** Brandon Lim (beml201@exeter.ac.uk)

**Background and Aims:** Thyroid hormones play a critical role in fetal growth, but their contribution to normal-range birthweight variation is not well-defined. A previous study found no evidence that maternal thyroid hormones affected offspring birthweight, but fetal hormones were not studied. Higher umbilical cord FT4 (ucFT4) was associated with higher birthweight in 616 UK babies; whether this reflects an effect of fetal thyroid hormones on fetal growth is unknown. We tested the causal effect of ucFT4 and umbilical cord TSH (ucTSH) on birthweight using Mendelian randomization (MR).

**Methods:** We performed 2-sample MR using single-nucleotide polymorphism (SNP) associations with FT4 (31 SNPs) and TSH (58 SNPs) from a genome-wide association study of adults (GWAS; n=72,167; sample1). We extracted corresponding SNP associations from GWAS of birthweight (n=406,063; sample2) where fetal genetic effects were adjusted for maternal-fetal genotype correlation. We estimated causal effects of ucFT4 and ucTSH on birthweight using inverse-variance weighted method. We checked whether genetic scores (GS) combining FT4 or TSH SNPs were associated with ucFT4 or ucTSH levels, respectively (n=669 babies).

**Results:** A 1SD higher fetal GS for FT4 was associated with a 0.12SD higher ucFT4 [95%CI: 0.04, 0.20] (P=0.002), but there was no evidence of a causal effect of ucFT4 (6g change in mean birthweight [95%CI:-9,21] per 1SD higher FT4; P=0.47). The GS for TSH was not associated with ucTSH (P=0.22), and there was no evidence that ucTSH influenced birthweight (P=0.59). Sensitivity analyses showed consistent results.

**Conclusions:** We found little evidence to support a causal relationship between ucFT4 or ucTSH levels and normal-range birthweight. The 95% confidence interval around the effect estimate excluded the previously reported observational association (110g higher birthweight per 1SD higher ucFT4). To verify instrument relevance, further work is necessary to check SNP effects on cord thyroid hormones are consistent with those from the original adult GWAS.

## OR5 Does chronic inflammation drive relapse in Graves’ disease?

### L C Lane^1,2^, T Cheetham^1,2^, S Razvi S^1,3^, S Pearce^1,4^

#### ^1^Translational and Clinical Research Institute, Newcastle University, Newcastle-Upon-Tyne, UK; ^2^Department of Paediatric Endocrinology, The Great North Children's Hospital, Newcastle-Upon-Tyne, UK; ^3^Queen Elizabeth Hospital, Gateshead Health NHS Foundation Trust, Gateshead, UK; ^4^Endocrine Unit, Royal Victoria Infirmary, Newcastle-Upon-Tyne, UK

##### **Correspondence:** Laura Lane (laura.lane@newcastle.ac.uk)

**Background and Aims:** Relapse in Graves’ disease (GD) is frequent after discontinuing antithyroid drug (ATD) treatment. The neutrophil- (NLR), monocyte- (MLR), and platelet- to lymphocyte ratio (PLR) have been proposed as biomarkers of inflammation and autoimmune disease activity. The purpose of this study was to evaluate whether the NLR, MLR, and PLR could be used as prognostic markers for predicting relapse in GD patients after ATD treatment.

**Methods:** This observational cohort study included 65 patients with GD who were followed-up for 12 months after stopping ATD. The NLR, MLR and PLR values were investigated at the time of stopping ATD and 6-8 weeks later. Disease outcome and relapse-free survival (RFS) after 12 months follow-up was evaluated. Receiver operating characteristic curve analysis was used to determine the optimal cut-off levels to differentiate disease outcome.

**Results:** Disease relapse within 12 months after ATD withdrawal occurred in 16 (25%) patients. In multivariate analysis, MLR at the end of ATD treatment was an independent prognostic factor for relapse (p=0.04) and time to relapse (p=0.01) in patients with GD after adjusting for age, sex, goitre, smoking status, thyroid hormone levels and thyrotropin receptor antibody titre. Patients with a high MLR (≥0.33) at the end of ATD treatment relapsed earlier (112.5 vs 311 days) and had poorer RFS than those with a low MLR (p = 0.03). C-reactive protein was positively correlated with monocyte count at both timepoints (p=0.01, p=0.02). There was no association with NLR and PLR and outcome in GD.

**Conclusions:** Elevated MLR may represent an independent prognostic biomarker for predicting relapse in GD. Chronic inflammation may therefore be associated with relapse following ATD withdrawal, thus further investigation to validate the role of this widely available, cost-effective inflammatory marker in the prognosis of GD is warranted.

## OR6 Structure of Full length TSH Receptor bound to TSH Receptor Blocking Monoclonal Autoantibody K1-70TM solved by Cryo-electron Microscopy

### R Nunez Miguel, P Sanders, L Allen, M Evans, M Holly, W Johnson, A Sullivan, J Miller-Gallacher, J Sanders, J Furmaniak, B Rees Smith

#### FIRS Laboratories, RSR Ltd, Parc Ty Glas, Llanishen, Cardiff, CF14 5DU, UK

##### **Correspondence:** J Furmaniak (firs@rsrltd.eclipse.co.uk)

**Background and Aims:** Structures of the TSH receptor (TSHR) leucine rich repeat domain (LRD) have been solved by crystallography and we now describe the use of cryo-electron microscopy (cryo-EM) to determine the structure of full length TSHR.

**Methods:** A complex of full length TSHR and monoclonal autoantibody K1-70TM was produced. TSHR expressed in CHO cells was incubated with K1-70TM Fab, the complex solubilised in 10mM Tris pH7.5, 50mM NaCl, 0.5g/L NaN3, 2% LMNG, 0.2% CHS and purified to homogeneity. Cryo-EM was performed on a Titan Krios 300kV with a K3 Direct Electron Detector.

**Results:** The TSHR- K1-70TM structure was solved to a global resolution of 3.3Å. A model was built using the previously solved TSHR LRD crystal structure and the AlfaFold TSHR model.

In the structure, full length TSHR is visible as a monomer with all three domains: LRD, hinge region (HR) and transmembrane domain (TMD) present. K1-70TM clasps the TSHR LRD as seen in the crystal structure. The TSHR extracellular domain (ECD) is composed of the LRD and HR in a similar arrangement to that in the crystal structure of the FSHR ECD and in the cryo-EM structure of the LH/CGR. The TSHR ECD is placed on top of the extracellular surface of the TMD in a similar orientation to the ECD in the structure of the LH/CGR inactive state. The HR forms interactions with the extracellular parts of the TMD. The structure and spatial positions of the TMD helices are similar in the TSHR and LH/CGR structures except for a 6.5Å displacement of the TSHR extracellular end of helix 6 compared to the LH/CGR inactive state structure.

**Conclusions:** The structure of full length TSHR in complex with K1-70TM has been solved and provides an excellent basis for understanding the mechanisms of action of TSHR autoantibodies and TSH.

## PO1 Promotion of thyroid cancer cell migration and invasion by the proto-oncogene PBF is mediated by FGD1 and N-WASP

### Mohammed Alshahrani, Selvambigai Manivannan, Caitlin EM Thornton, Katie Brookes, Saroop Raja, Hannah R Nieto, Martin L Read, Christopher J McCabe & Vicki E Smith

#### Institute of Metabolism and Systems Research, University of Birmingham, Birmingham, UK

##### **Correspondence:** Selvambigai Manivannan (s.manivannan@bham.ac.uk)

**Background:** Cell motility is a highly complex process that involves the co-ordination of cell adhesion, actin dynamics and signal transduction. The proto-oncogene pituitary tumor-transforming gene (PTTG)-binding factor (PBF/PTTG1IP) is a ubiquitously expressed transmembrane glycoprotein that promotes cellular migration and invasion through phosphorylation at PBF-Y174 by Src kinase. Alterations in the phosphoproteome following PBF overexpression in normal thyroid epithelial cells (Nthy-ori 3-1) show an enrichment of proteins involved in cytoskeletal arrangement, cell adhesion and small GTPase activity. FYVE, RhoGEF and PH domain-containing protein 1 (FGD1) and Neural Wiskott-Aldrich syndrome protein (N-WASP) phosphorylation was significantly altered with PBF upregulation.

**Objective:** Given the involvement of FGD1 and N-WASP in small GTPase signalling and cell motility we investigated a role for FGD1 and N-WASP in PBF-induced motility of TPC-1 thyroid cancer cells.

**Methods:** For FGD1 and N-WASP gene knock down, RNA interference (RNAi or siRNA) was performed. Effects of FGD1 and N-WASP on cell motility and invasion was evaluated by scratch wound migration and transwell invasion assays, respectively.

**Results:** siRNA-mediated knockdown of either FGD1 or N-WASP significantly abrogated both PBF-induced cell migration and invasion. Co-expression of either FGD1 or N-WASP with PBF did not further stimulate cell invasion. However, data suggest there may be a combined effect of PBF and N-WASP overexpression on cell migration.

**Conclusions:** Taken together, these preliminary findings suggest that both FGD1 and N-WASP mediate the induction of cell motility by PBF in thyroid cancer cells, revealing novel signalling events in thyroid cancer progression.

## PO2 PBF phosphorylation regulates thyroid cancer cell motility

### Merve Kocbiyik, Mohammed Alshahrani, Vikki L Poole, Selvambigai Manivannan, Caitlin Thornton, Ling Zha, Katie Brookes, Hannah Nieto, Martin L Read, Chris J McCabe, Vicki E Smith

#### Institute of Metabolism and Systems Research (IMSR), University of Birmingham, Birmingham, UK

##### **Correspondence:** Merve Kocbiyik (mxk1003@student.bham.ac.uk)

**Background and Aims:** The proto-oncogene pituitary tumour transforming gene binding factor (PTTG1IP/PBF) is overexpressed in multiple tumours, including thyroid cancer, and is associated with tumour progression. PBF mediates several tumourigenic processes, such as cell motility, and induces thyroid cancer cell invasion. However, in contrast to wild-type (WT) PBF, PBF-Y174A is unable to induce cell invasion. The Y174 residue is highly phosphorylated and this suggests that phosphorylation mediates PBF-induced thyroid cancer cell invasion. The aim of these studies was to further elucidate PBF regulation of cell motility.

**Methods:** Several PBF mutants were utilised. Y174A substitution also causes plasma membrane retention due to disruption of an endocytosis motif. Thus, to better understand the impact of PBF phosphorylation and localisation on cell motility, a mutant with a disrupted Src consensus sequence (EEN170-172AAA;‘PBF-EEN’) and another with a substitution at F177 (F177A) were also employed. PBF-EEN shows largely vesicular localisation, similar to WT PBF, but with reduced phosphorylation. In contrast, F177A accumulates at the plasma membrane due to endocytosis motif disruption but is still phosphorylated. Cell motility was determined using scratch wound, Transwell migration and Transwell invasion assays.

**Results:** As shown previously, PBF overexpression resulted in significant induction of TPC1 thyroid cancer cell migration using both scratch wound and Transwell migration assays. In contrast, neither Y174A, PBF-EEN nor F177A were able to stimulate cell migration. Similarly, whilst WT PBF significantly induced TPC1 cell invasion, none of the 3 mutants had any effect. These results were replicated in the MCF7 breast cancer cell line demonstrating their relevance to other tumour types.

**Conclusion:** These findings demonstrate that PBF phosphorylation is critical for PBF induction of cell motility and also suggest that PBF endocytosis is essential. This study provides more insight into the mechanism of PBF-regulated cell motility and supports PBF-pY174 as a potential therapeutic target in thyroid cancer.

## PO3 AP-2 and PBF Regulate the Internalisation of the Sodium-Iodide Symporter (NIS)

### Ling Zha, Kate Brookes, Caitlin E M Thornton, Alice Fletcher, Martin L Read, Vicki E Smith, Merve Kocbiyik, Selvambigai Manivannan, Marie Christine Jones, Christopher J McCabe

#### Institute of Metabolism and Systems Research, University of Birmingham, Birmingham, UK

##### **Correspondence:** Ling Zha (lxz027@student.bham.ac.uk)

**Background:** Sodium/iodide symporter (NIS) expression is frequently downregulated and/or shows diminished targeting to the plasma membrane in differentiated thyroid cancer, resulting in suboptimal radioiodine treatment and poor prognosis. The mechanisms which govern the endocytosis of NIS away from the plasma membrane (PM) – its sole site of transport activity – are ill-defined and may be of direct therapeutic potential in enhancing radioiodine treatment. We previously showed that the proto-oncogene PTTG1-binding factor (PBF) binds NIS and enhances its internalisation, hypothesising that this was via clathrin and adaptor protein complex 2 (AP2) mediated mechanisms. We now challenge this hypothesis experimentally.

**Aims:** To better understand the role of AP2 and PBF in NIS internalisation, as well as the functionality of newly identified putative endocytosis motifs within the NIS C-terminus.

**Methods:** Putative endocytosis motifs were ameliorated via site-directed mutagenesis. We employed 125I radioiodine uptake assays to test the function of wild type and mutated NIS constructs. The AP2 subunits μ2, σ2 and ⍺1 were ablated via siRNA. The stringency of NIS and PBF binding was determined via NanoBiT assays.

**Results:** We first identified a putative acidic dipeptide located within the NIS C-terminus at E578-E579. Transfection of NIS with a mutated acidic dipeptide (E578A/E579A) resulted in significantly increased 125I uptake compared to wild-type NIS in HeLa cells (1.5-fold, N=4, P<0.05), and retention of NIS protein at the PM, as determined by immunofluorescent microscopy. Transient siRNA knockdown of the AP2 subunits ⍺1 and μ2 significantly increased NIS and PBF binding in NanoBiT assays in HeLa cells (N=5, P<0.05).

**Conclusions:** We identify a diacidic motif in the C-terminus of NIS which impacts NIS localisation and function. NanoBiT protein-protein interaction assays confirmed measurable interaction between NIS and PBF, which was significantly enhanced via knockdown of AP2 subunits, suggesting that AP2 plays a central role in NIS endocytosis.

## PO4 Is repeat fine needle aspiration required in thyroid nodules with initial benign cytology? Results from a large Irish series

### HM Zia-ul-Hussnain^1^, O Kgosidialwa^1^, Kennedy C^1^, M Quinn^1^, E Dolan^1^, P Deignan^1^, M Sherlock^1^, CJ Thompson^1^, D Smith^1^, JP O’Neill^2^, A Hill^2^, M Leader^3^, H Barrett^3^, C Ryan^3^, Keeling F^4^, MM Morrin^4^, A Agha^1^

#### ^1^Academic Department of Endocrinology, Beaumont Hospital, Dublin, Ireland; ^2^Department of Surgery, Beaumont Hospital, Dublin, Ireland; ^3^Department of Histopathology, Beaumont Hospital, Dublin, Ireland; ^4^Department of Radiology, Beaumont Hospital, Dublin, Ireland

##### **Correspondence:** Oratile Kgosidialwa (oratile.kgosidialwa2@hse.ie)

^*^Both authors had an equal contribution to the manuscript

**Background**: Fine needle aspiration (FNA) cytology is the preferred method for assessing thyroid nodules for malignancy. Concern remains about the rate of false negative results. The primary aim of this study is to investigate the malignancy rate of thyroid nodules initially classified as benign (Thy 2).

**Methods**: We retrospectively examined 658 nodules in 653 (429 female) patients between January 2013 to December 2017. All FNA biopsies (FNABs) were performed under ultrasound (US) guidance by a radiologist with expertise in thyroid pathology. Nodules were cytologically classified according to the UK Royal College of Pathologists guidelines. Decisions about further management were made at a regular thyroid multidisciplinary meeting. Follow up of the Thy 2 nodules was determined based on clinical and radiological criteria.

**Results**: The mean age (±SD) was 53.2 (14.6) years. Five hundred out of 658 (76.0%) nodules were classified as Thy 2 (benign) after the first FNAB. Of these thyroid nodules initially classified as benign, 208 (41.6%) underwent repeat FNAB and 9 (1.8%) were surgically removed without repeat FNAB. The remainder were followed up clinically and/or radiologically. Seven (1.4%) of nodules initially classified as Thy 2 were later shown to be or to harbor malignancy after a follow-up period of 74.5 (±19.7) months. Papillary thyroid microcarcinomas were found co-incidentally in two thyroid glands of benign nodules, giving a more meaningful false negative rate of 1%.

**Conclusions**: With a well-targeted FNAB, the false negative rate of an initial benign thyroid FNA is very low, thus routine second FNAB is not required in patients with a thyroid nodule initially deemed benign. Multidisciplinary input is imperative in informing decision-making.

## PO5 Discordant thyroid function tests following radioactive iodine administration for suspected Graves’ Disease – a diagnostic challenge

### M Lockhart^1^, C Moran C^2,3,4^, S Sreenan^1,5^

#### ^1^Academic Department of Endocrinology and Diabetes, Connolly Hospital Blanchardstown, Dublin 15, Ireland; ^2^Endocrine & Diabetes Section, Beacon Hospital, Sandyford, Dublin 18, Ireland; ^3^St Vincent’s University Hospital, Dublin 4, Ireland; ^4^School of Medicine, University College Dublin, Ireland; ^5^Royal College of Surgeons Ireland, Ireland

##### **Correspondence:** Michael Lockhart (lockhamj@tcd.ie)

**Background:** The majority of thyroid function tests results are straightforward to interpret, but occasionally unusual patterns arise, which can cause significant diagnostic difficulty.

**Case Presentation:** A 22-year-old female was referred to the Endocrinology clinic with an eight-month history of amenorrhoea following the birth of her first child. She was noted to have elevated FT4 of 33pmol/L (12 – 30pmol/L) with a normal TSH of 1.0mIU/L (0.45 – 4.5mIU/L). Repeat testing showed a mildly suppressed TSH (0.05mIU/L) with an elevated FT4 (33pmol/L). She was clinically euthyroid, had a small goitre on examination and had no evidence of thyroid eye disease. Anti-TRAb and anti-TPO Ab were negative. Carbimazole therapy was commenced. TFTs over the following years showed a persistent pattern of raised FT4 and normal TSH despite dose adjustment of carbimazole. Ultrasound imaging of the thyroid gland was unremarkable. A radioactive iodine uptake scan showed homogenous uptake, felt to support the diagnosis of antibody-negative Graves’ disease. RAI treatment was administered six years after presentation, given the persistently abnormal TFTs despite medical therapy. After this, FT4 levels remained elevated but now with a raised TSH – levothyroxine was commenced. Levothyroxine dose was adjusted several times as the TSH intermittently rose above the reference range, while FT4 remained persistently elevated. Samples sent to an external laboratory with a two-step immunoassay revealed a similar pattern. It came to light that the patient had a family history of “hyperthyroidism” in her mother. Maternal TFTs, and later those from the patient’s 20-year-old daughter, revealed raised FT4 and normal TSH. Radioligand binding assay confirmed a biochemical diagnosis of Familial Dysalbuminaemic Hyperthyroxinaemia, and ALB gene sequencing is in process.

**Conclusion:** This case highlights the importance of confirming persistent abnormalities in TFT results prior to administration of treatment for hyperthyroidism, and also that discordant TFTs should prompt further investigation to identify a potential genetic disorder, regardless of prior diagnosis and treatment.

The patient gave written consent to this publication.

TFT = Thyroid Function Test

FT4 = Free Thyroxine

TSH = Thyroid-Stimulating Hormone

TRAB = TSH Receptor Antibodies

Anti-TPO Ab = Anti-Thyroid Peroxidase Antibodies

US = Ultrasound

RAI = Radioactive Iodine

## PO6 Funny thyroid function tests - TSHoma and post-COVID 19 thyroiditis leading to confusing thyroid function tests dynamics, a case report

### M Lockhart, T McDonnell, D Smith, M Javadpour, A Agha

#### Department of Endocrinology, Beaumont Hospital, RCSI, Dublin, Ireland; Department of Neurosurgery, Beaumont Hospital, RCSI, Dublin, Ireland

##### **Correspondence:** M Lockhart (lockhamj@tcd.ie)

**Background:** We present the case of a male patient with a diagnosis of TSH-secreting pituitary adenoma (TSHoma) who became infected with SARS-CoV-2 and developed a post-COVID-19 thyroiditis with resultant confusing changes in his thyroid function tests (TFTs).

**Case Presentation:** The patient presented with an incidental finding of elevated Free T4 and inappropriately normal TSH, confirmed on multiple analytical platforms. A TRH test showed a flat TSH response, and an MRI pituitary showed a 2.4 cm macroadenoma. Somatostatin analogue treatment was commenced pending surgery, with rapid normalization of TFTs.

The patient then became symptomatic of headache, pyrexia, dysgeusia and anosmia lasting two weeks, at a time when the first wave of the COVID-19 pandemic was affecting Ireland. The patient had been a close contact of two confirmed COVID-19 cases. He did not have a SARS-CoV2 PCR test at the time but later tested positive for COVID-19 spike and nucleocapsid antigen IgG antibodies (vaccine naïve), indicating previous exposure to SARS-CoV-2. Two months after this illness, the patient’s TFTs showed a pattern typical of primary hyperthyroidism with grossly elevated FT4 and fully suppressed TSH (with co-existent thyrotoxicosis symptoms), followed by a pattern of primary hypothyroidism with a low FT4 and high TSH – a pattern consistent with subacute thyroiditis post-viral illness. TRAb was negative.

The patient’s TFTs later showed high normal TSH and normal FT4 while continuing lanreotide therapy. He is currently euthyroid and awaiting pituitary surgery which was delayed due to the COVID-19 emergency.

**Conclusion:** To our knowledge, this is the first case of post-COVID-19 thyroiditis in a patient with underlying TSHoma. The case highlights the importance of considering an alternative or new diagnosis in the setting of rapidly changing patterns in thyroid function tests, and for close clinical and biochemical follow-up in these situations.

The patient gave written consent to this publication.

SARS-CoV-2 = Severe Acute Respiratory Syndrome Coronavirus 2

TFT = Thyroid Function Test

FT4 = Free Thyroxine

TSH = Thyroid-Stimulating Hormone

TRH = Thyrotropin-Releasing Hormone

MRI = Magnetic Resonance Imaging

TRAB = TSH Receptor Antibodies

Anti-TPO Ab = Anti-Thyroid Peroxidase Antibodies

## PO7 Graves’ thyrotoxicosis contributing to post-partum cardiogenic shock in a patient with undiagnosed cardiomyopathy

### R M Flynn^1^, K Gunganah^1^, W M Drake^2^

#### ^1^Department of Diabetes and Endocrinology. Newham University Hospital, UK; ^2^Department of Diabetes and Endocrinology. St Barthlomomew’s hospital, London, UK

##### **Correspondence:** Rachel Flynn (rachel.flynn5@nhs.net)

**Case Presentation:** A 22-year-old primiparous woman presented at 32 weeks gestation with pre-eclampsia and pre-term labour. She underwent an emergency C-section at 33+1 weeks and suffered a cardiac arrest post-delivery. She was transferred to ITU following return of spontaneous circulation and required ventilatory support, magnesium infusion and antihypertensive therapy. She was also found to be hypoxic due to COVID-19 pneumonitis and was started on Dexamethasone and antibiotics. She made a rapid and uneventful recovery. Prior to discharge, she was noted to be thyrotoxic with a Free T4 of 75.3 pmol/L and TSH of less than 0.01 mU/L. She was started on Carbimazole and Prednisolone (to cover for COVID-related thyroiditis). She remained on Labetalol for hypertension management. Outpatient endocrinology follow-up was arranged. Six weeks after discharge, she re-presented moribund in cardiogenic shock and was transferred to a cardiac centre.

**Investigations:** Free T4 > 100 pmol/L, TSH < 0.01 mU/L, TSH receptor Ab 3.33 iU/L. NM thyroid uptake scan: symmetrical and increased uptake consistent with Graves’ disease. CXR: Cardiomegaly with upper lobe diversion. Echocardiogram: Severely dilated left ventricle with severe global dysfunction, increased pulmonary artery pressure and LV thrombus. Cardiac MRI: Moderate-to-severe dilatation of all cardiac chambers with hypertrophy, indicating a degree of chronicity.

**Progress:** She was intubated and ventilated and started on Propylthiouracil, Esmolol and Hydrocortisone infusion. She was also started on prognostic heart failure medication and anticoagulation therapy. She made a good recovery and her thyroid function tests improved rapidly (Free T4 29.2 pmol/L and TSH < 0.01 mU/L) within 1 week of admission. She was weaned off steroids and switched from Propylthiouracil to Carbimazole on discharge.

**Conclusion:** The working diagnosis is that of a multifactorial post-partum cardiomyopathy worsened by concurrent uncontrolled thyrotoxicosis. It is likely that immunomodulation during pregnancy was “protective” against Graves’ thyrotoxicosis and worsened post-partum.

Written consent to publish had been obtained from the patient.

## PO8 Refractory Graves’ disease dramatically responded to adjunctive cholestyramine, a case report and literature review

### Mohammad Bilal Jajah, Heng Chun Wong, Jayamalee Jayaweera

#### Diabetes and Endocrinology Department, West Suffolk Hospital NHS Foundation Trust, Bury St Edmunds IP33 2QZ, UK

##### **Correspondence:** Mohammad Bilal Jajah (drmbilal77@hotmail.com)

**Background:** Graves’ disease usually responds well to medical treatment with thionamides. However, in some cases, it fails to respond to this treatment, even at maximum doses. A few reported cases have shown that cholestyramine helps to restore normal thyroid function when added to the ongoing anti-thyroidal medications in refractory thyrotoxicosis. We report a case of relapsing refractory Graves’ disease, in which cholestyramine has helped to restore normal thyroid function tests and allowed for subsequent total thyroidectomy. We discuss three other published cases of refractory thyrotoxicosis which responded well to adjunct cholestyramine therapy.

**Case Presentation:** A 21-year-old woman presented with relapsing Graves’ disease after 5 years of remission. She was planned for surgery and started on carbimazole in order to restore a euthyroid state before the procedure. This was not achieved despite carbimazole doses being increased to 60 mg over a period of 8 weeks. Cholestyramine, 4 mg four times a day, was added to her treatment regimen, alongside increasing her dose of propranolol. Marked changes were noted in her thyroid function tests after less than three weeks into this treatment regimen showing FT4 and FT3 falling toward the normal ranges. The patient subsequently underwent successful total thyroidectomy.

**Discussion:** Cholestyramine has been found to reduce thyroid hormone levels in patients with thyrotoxicosis by interfering enterohepatic circulation and recycling of thyroid hormone. A few case reports have noted that cholestyramine, when added to antithyroid drugs in patients with refractory thyrotoxicosis, has successfully achieved a euthyroid state within a few weeks of treatment. Our case further supports that cholestyramine could be used as an adjunct in treating this group of patients.

**Conclusion:** Cholestyramine could be an effective additional treatment in refractory thyrotoxicosis when maximum doses of thionamides fail to restore normal thyroid function.

Written consent to publish had been obtained from the patient.

## PO9 Techniques to minimize post-operative complications in thyroid surgery

### Abdulwahid M. Salih^1,2^, Ari M. Abdullah^1,4^, Aras J. Qaradakhy^1,5^, Hiwa O. Baba^1,2^, Shko H. Hassan^1^, Saeed Karim^1^, Fahmi H. kakamad^1,2,3^, Zuhair D Hammood^1^, Yadgar A. Saeed^1^, Aso S. Muhialdeen^1,2^, Hardi M. Dhahir^1,6^, Hallkawt O. Ali^1,8^, Rebaz O. Muhammed^1^, Nawshirwan H. Abdulkarim^8^, Kayhan A Najar^1,2^

#### ^1^Smart Health Tower, Madam Mitterrand Street, Sulaimani, Kurdistan, Iraq; ^2^Kscien Organization, Hamdi Str, Azadi Mall, Sulaimani, Kurdistan, Iraq; ^3^College of Medicine, University of Sulaimani, Madam Mitterrand Street, Sulaimani, Kurdistan, Iraq; ^4^Sulaimani Teaching Hospital, Sulaimani, Kurdistan, Iraq; ^5^Shorsh teaching hospital, Sulaimani, Kurdistan, Iraq; ^6^Shahid Peshraw Hospital, Sulaimani, Kurdistan, Iraq; ^7^Shahidan Qaladze Teaching Hospital, Qaladze, Kurdistan, Iraq; ^8^Kalar General Hospital, Kalar, Kurdistan, Iraq

##### **Correspondence:** Abdulwahid Mohammed Salih (Abdulwahid.salih@univsul.edu.iq)

**Objective:** To report the use of four techniques applied in patients undergoing total and hemithyroidectomy over the course of four years that have shown to decrease the rate of postoperative hypocalcemia and preserve recurrent laryngeal nerve while having a good cosmetic outcome.

**Methods:** The techniques included (1) elevation of a circular flap after 4 cm collar incision, (2) ligation of the pedicles (middle first-vein, middle last-artery), (3) sharp dissection of the parathyroid glands, and (4) sharp and blunt dissection and exposure of the nerve in all of the patients.

**Results:** The total number of patients was 2399, ages ranging from 10 to 89 years with a mean age of 44.7 years. Neck swelling was the most common presenting symptom (844, 35.2%). At least one symptom of hyperthyroidism was found in 1271 patients (53%). The main indications for the operation were: multinodular goitre and compressive symptoms in 1331 (55.5%) patients, thyrotoxicosis in 598 (25%) patients and malignancy in 402 (16.8%) patients. The most common operation was total thyroidectomy in 1880 (78.4%) patients, followed by thyroid lobectomy in 495 (20.6%) patients and completion thyroidectomy in 24 (1%) patients. Among all patients, 4 (0.16%) patients developed permanent voice change. Seven (0.3%) patients developed permanent hypocalcemia.

**Conclusions:** Thyroidectomy is a relatively safe procedure with limited serious complications. Even with new emerging techniques risks still remain. The presenting study offers a set of surgical techniques that reduce complication risks after thyroidectomy.

## PO10 Simultaneous triple pathology in the thyroid gland with primary Hodgkin lymphoma, a case report

### Ari M. Abdullah^1,4^, Abdulwahid M. Salih^1,2^, Aras J. Qaradakhy^1,5^, Shko H. Hassan^1^, Saeed Karim^1^, Fahmi H. kakamad^1,2,3^

#### ^1^Smart Health Tower, Madam Mitterrand Street, Sulaimani, Kurdistan, Iraq; ^2^Kscien Organization, Hamdi Str, Azadi Mall, Sulaimani, Kurdistan, Iraq; ^3^College of Medicine, University of Sulaimani, Madam Mitterrand Street, Sulaimani, Kurdistan, Iraq; ^4^Sulaimani Teaching Hospital, Sulaimani, Kurdistan, Iraq; ^5^Shorsh Teaching Hospital, Sulaimani, Kurdistan, Iraq

##### **Correspondence:** Abdulwahid Mohammed Salih (Abdulwahid.salih@univsul.edu.iq)

**Background:** Coexistence of two or three different tumours in the thyroid gland is an extremely rare finding. The aim of this report is to present a case of papillary thyroid carcinoma, hyalinizing trabecular tumour and Hodgkin’s lymphoma.

**Case presentation:** A 32-year-old female presented with an incidental finding of thyroid nodules on neck ultrasound. She presented with two highly suspicious nodules in the left side of the thyroid gland and bilateral multiple pathological lymph nodes. Fine-needle aspiration cytology suggested papillary thyroid carcinoma and Hodgkin’s lymphoma. The patient underwent total thyroidectomy with excisional biopsy of one of the cervical lymph nodes. Histopathological examination of the specimen revealed multifocal papillary microcarcinoma-conventional type and hyalinizing trabecular tumour in the left thyroid lobe in the setting of Hashimoto thyroiditis, with excisional biopsy from the cervical lymph node showed Hodgkin’s lymphoma.

**Conclusion:** Although it is extremely rare, triple pathology in the thyroid gland is possible.

Written consent to publish had been obtained from the patient.

